# Superior epigastric artery pseudoaneurysm- a rare complication of chest drain insertion in coronary artery bypass grafting

**DOI:** 10.1186/1749-8090-2-21

**Published:** 2007-04-25

**Authors:** Umar Sadat, Asif Jah, Nick Ward, Michael Gaunt

**Affiliations:** 1 Department of Surgery, Cambridge University Hospitals NHS Foundation Trust, Cambridge, UK

## Abstract

**Background:**

Although chest drain insertion during coronary artery bypass grafting is a fairly standard procedure, however it may result in extremely rare complications.

**Case presentation:**

This is the first case being reported that demonstrates a pseudoaneurysm of superior epigastric artery resulting from chest drain insertion following coronary artery bypass grafting.

**Conclusion:**

Adequate caution should be used along with good understanding of the anatomical landmarks during apparently simple and standard operative procedures.

## Background

This is the first case being reported that demonstrates a pseudoaneurysm of superior epigastric artery resulting from chest drain insertion in a patient undergoing coronary artery bypass grafting. Adequate caution should be used along with good understanding of the anatomical landmarks during such operative procedures.

## Case presentation

**Figure 1:**
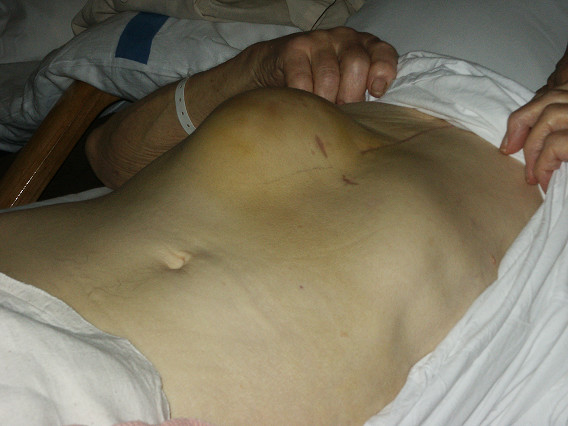
Swelling in the right hypochondrium, which on palpation revealed a pulsatile mass.

**Figure 2:**
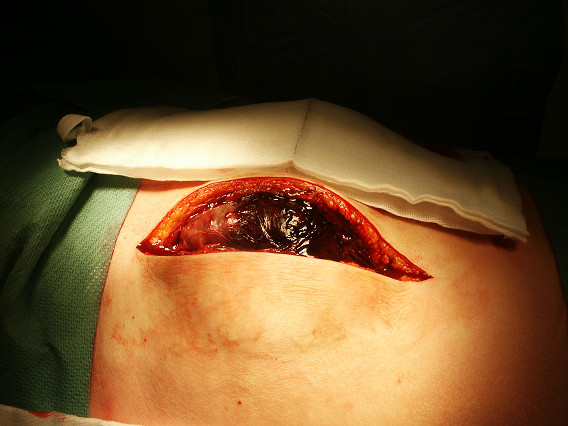
Large pseudoaneurysm walled off by the surrounding tissue.

A 65-year-old patient, who had undergone coronary artery bypass grafting (CABG) 7 days ago for triple vessel disease, presented in the emergency department with signs of hypovolemic shock and rapidly progressing swelling in the right upper quadrant of the abdomen (Figure 1). He was discharged from the hospital on the fourth postoperative day after CABG with no intraopertive and postoperative complications noted. This swelling was in the area of previously placed mediastinal chest drain in subxyphoid region during CABG, which was removed on second postoperative day. Initial resuscitatation was done by intravenous fluid administration and blood transfusions. Examination of the swelling demonstrated an expansile and pulsatile tender mass possibly resulting from trauma to superior epigastric artery (SEA). This was confirmed with a Doppler ultrasound scan as a pseudoaneurysm of SEA measuring 5 × 7 cm. Emergency surgical exploration was carried out and the pseudoaneurysm identified (Figure 2). The haematoma was evacuated and the defect in the artery was repaired primarily with prolene suture. His recovery was uneventful and was discharged from the hospital on fourth postoperative day.

## Discussion

Pseudoaneurysms result from injury to the blood vessel wall; the leaking blood later becomes walled off by surrounding tissue, the communication with the vessel making them pulsatile. Common causative factors include iatrogenic injury [1], trauma [2], infection (mycotic), vasculitic disease [3] etc.

Although investigative tools for pseudoaneurysms include doppler ultrasound scan or angiograms (digital subtraction angiogram or CT/MR angiograms), in this case the diagnosis was entirely clinical with confirmation by duplex scan. Invasive angiograms are usually performed when there is intent to treat the pseudoaneurysm with embolization. Pseudoaneurysms have a tendency to thrombose on their own, but mostly they require either surgical or radiological intervention. The latter involves ultrasound compression of the pseudoaneurysm, thrombin injection into the neck of the pseudoaneurysm or coil embolization. However, in large pseudoaneurysms presenting with shock, open surgical repair is done after initial resuscitation as in this case.

Literature search using medline search engine shows that it is the first case to be reported of superior epigastric artery pseudoaneurysm after chest drain insertion following coronary artery bypass graft. Other complications include damage to the lung parenchyma, injury to the pulmonary artery, blockage of the drain etc. Chest drains after CABG can be placed either in the intercostal region and/or the subxyphoid region. The latter has been shown to be less painful and associated with better post op chest physiotherapy [4].

To avoid such uncommon complications adequate anatomical knowledge is required during chest drain insertions. As insertion of chest drain is under direct vision; hence the risk of injury vessels is low. This case report appears to be an unfortunate and isolated case, which should not affect the surgeon's choice of the chest drain's site. Perhaps, it reminds us that Murphy's law is very much alive: *anything that can go wrong, will*. Hence, it may be added to the pre-op counselling of patients that the apparently innocuous chest drain can give trouble. In addition, as an added precaution, a check can be made to ensure that the drain is well away from the superior epigastric artery prior to closing the chest.

## Conflict of interests

The author(s) declare that they have no competing interests.

## Authors' contributions

All the authors have been involved in literature search, writing and final reviewing of this manuscript.
